# European Reference Networks: developing a EUCERD opinion

**DOI:** 10.1186/1750-1172-7-S2-A6

**Published:** 2012-11-22

**Authors:** Kate Bushby

**Affiliations:** 1 Institute of Genetic Medicine, International Centre for Life, Central Parkway, Newcastle upon Tyne, NE1 3BZ, United Kingdom

## 

The establishment of European Reference Networks as laid out under the Cross Border Health Care Directive (CBHCD) is a major opportunity for the rare disease community. There have been many successful networks for rare disease groups, but their establishment has been ad hoc and funding streams variable. Sustainability has been a major challenge. Although the assessment of the quality of these networks has not been systematic, nonetheless networks have succeeded in establishing important infrastructure including disease specific registries, shared tools such as tele-expertise and the production of disease specific guidelines and training pathways.

Within the CBHCD it is envisaged that European Reference networks will be established, and these will not only relate to rare diseases. These networks will primarily link nationally designated centres of expertise. Within the EUCERD a process is being followed to inform the CBHCD committee on the specific issues relating to ERNs for rare diseases. These recommendations relate to areas of designation and governance, capacity building and resources to support ERNs and quality assurance.

The recommendations will be discussed in a series of meetings in 2012, with the aim of producing a EUCERD recommendation on ERNs for rare diseases by the end of the year in line with the timeline of the cross border health care directive.

